# Brain Arteriovenous Malformations Treated With Linear Accelerator-Based Stereotactic Radiosurgery: A Single-Institution Retrospective Experience From Latin America

**DOI:** 10.7759/cureus.109975

**Published:** 2026-05-31

**Authors:** Valentina Rangel-Sarmiento, Beatriz Soto-Cala, Juanita Cure, Maria Caicedo-Martinez, German Borda, Carlos A Lindado, Esperanza Castro-Lombana, Juan C Puentes, Oscar Zorro, Edwin O Pulido-Ramirez, Alejandro Gonzalez-Motta

**Affiliations:** 1 Oncology Research Group, Faculty of Medicine, Pontificia Universidad Javeriana, Bogota, COL; 2 Javeriana Oncology Center, Hospital Universitario San Ignacio, Bogota, COL; 3 Cancer Epidemiology Research Program, L'Hospitalet de Llobregat, Catalan Institute of Oncology, Institut d'Investigació Biomèdica de Bellvitge (IDIBELL), Barcelona, ESP; 4 Radiation Oncology Department, Instituto Nacional de Cancerología, Bogota, COL; 5 Neurosurgery Department, Hospital Universitario San Ignacio, Bogota, COL; 6 Institute for Research, Science, and Education, GIGA Research Group, Luis Carlos Sarmiento Angulo Cancer Treatment and Research Center (CTIC), Bogota, COL; 7 Radiotherapy Functional Unit, Luis Carlos Sarmiento Angulo Cancer Treatment and Research Center (CTIC), Bogota, COL

**Keywords:** brain arteriovenous malformations, latin america, linear accelerator, radiosurgery outcomes, stereotactic radiosurgery, vascular malformations

## Abstract

Background

Stereotactic radiosurgery (SRS) is an established treatment option for selected patients with brain arteriovenous malformations (bAVMs), particularly for lesions not amenable to microsurgical resection or located in surgically high-risk areas. SRS can be delivered through different platforms, including Gamma Knife, CyberKnife, and linear accelerator (LINAC)-based systems. This retrospective single-institution study evaluated outcomes of patients with bAVMs treated with LINAC-based SRS at a quaternary care center in Bogotá, Colombia.

Materials and methods

We conducted a retrospective observational study of patients with bAVMs treated with LINAC-based SRS guided by triple-fusion imaging between 2011 and 2017. Clinical, treatment, and imaging data were collected from medical records. Telephone interviews were used only to supplement clinical follow-up and were not used to determine radiological obliteration. The primary outcome was radiological obliteration during the available follow-up period, assessed by follow-up imaging. Actuarial obliteration rates were estimated using Kaplan-Meier analysis and interpreted as exploratory because of limited follow-up and censoring. Univariable Cox proportional hazards analyses were performed as exploratory analyses to evaluate factors associated with obliteration.

Results

Eighty-two patients were included. At presentation, 36 patients (44.0%) had ruptured bAVMs, 61 (74.4%) had lesions in eloquent areas, and most had Spetzler-Martin grade III malformations. Prior embolization was performed in 47 patients (57.3%). Complete radiological obliteration was documented in 27 patients (33.0%) during available follow-up. The median follow-up was 16.3 months, and 46 patients (56.1%) were lost to follow-up before completing three years of institutional follow-up. Actuarial obliteration estimates at later time points should therefore be interpreted cautiously. In exploratory univariable Cox analysis, radiosurgery dose >20 Gy was associated with a shorter time to obliteration.

Conclusions

This single-institution retrospective study describes radiological and clinical outcomes after LINAC-based SRS for bAVMs in a Latin American quaternary care center. The findings suggest that LINAC-based SRS is a feasible treatment approach when appropriate technology, planning expertise, and quality assurance are available. However, the short median follow-up, substantial loss to follow-up, and lack of a comparator group limit conclusions regarding effectiveness. Future prospective studies with standardized long-term imaging follow-up are needed.

## Introduction

Brain arteriovenous malformations (bAVMs) are the most common intracranial vascular malformation [1,2], consisting of an aberrant communication between arterial and venous systems [3]. They are often diagnosed due to symptoms from intracranial hemorrhage, which accounts for 1-2% of strokes in the general population [2]. Rupture risk is influenced by demographic (advanced age [4], Hispanic ethnicity [5]), clinical (prior bleeding [6]), and anatomical factors (location, deep venous drainage, associated aneurysms [6,7]). Grading systems, such as the Spetzler-Martin (SM) grading system, help predict treatment outcomes [8].

Treatment aims for complete bAVM obliteration as prophylaxis against spontaneous intracranial hemorrhage. It includes conservative and invasive measures, such as microsurgery, embolization, stereotactic radiosurgery (SRS), or combinations thereof. Treatment decisions depend on SM grading, bleeding risk [8,9], available resources, and institutional expertise [10]. Partial embolization may precede other treatments; however, it has been linked to increased infarction risk, lower SRS efficacy [11], and minimal monotherapy benefit on long-term hemorrhagic risk [12]. Thus, it is usually reserved as a palliative measure for high-grade bAVMs [13].

Microsurgery is preferred for low-grade bAVMs, with over 96% obliteration rates [9,14]. Nonetheless, due to surgical morbidity and risk to surrounding tissue, SRS is used as a non-invasive alternative, though its indications remain debated [15,16]. SRS is an outpatient, image-guided procedure that delivers ionizing radiation to the nidus of the bAVM. Obliteration rates range from 60% to 80% [4,15]. After obliteration, hemorrhage risk drops below 1%. A key limitation is the latency period (two to four years), during which bleeding can still occur. Additionally, precise techniques are required because radiation to adjacent structures can cause cerebral edema and necrosis [17].

Evidence of SRS for bAVM management is limited. The only clinical trial (ARUBA) [18] was criticized [15], and most of the literature on SRS for bAVMs focuses on Gamma Knife and Cyber Knife [17,19-26], which are often unavailable in lower-resource countries such as Colombia. This evidence makes linear accelerator (LINAC)-based SRS appealing, showing comparable obliteration and safety outcomes [27-30]. However, evidence is scarce in our context. Our study aimed to describe radiological obliteration outcomes, clinical follow-up, and exploratory factors associated with obliteration after LINAC-based SRS for bAVMs in a single Latin American quaternary care institution.

This manuscript was previously posted as a preprint on the Research Square server on November 26th, 2024, and on SSRN on July 8, 2025.

## Materials and methods

Study design and data collection

We conducted an observational study of bAVM patients treated with LINAC-based SRS using triple-image fusion (magnetic resonance imaging (MRI), computed tomography (CT), and angiography) at our institution between 2011 and 2017. Patients were referred after a neurosurgical evaluation deemed SRS to be the most appropriate. Exclusion criteria included patients younger than 14 years or those with brain cancer. After approval from the Research and Institutional Ethics Committee of the Faculty of Medicine of the Pontificia Universidad Javeriana and Hospital Universitario San Ignacio (approval number: 2020/179), data were collected retrospectively from medical records and through phone follow-ups. Data were entered in REDCap® [31,32].

The primary outcome was radiological obliteration during the available follow-up period, defined as the absence of a residual nidus on follow-up angiography or MRI. When both modalities were available, angiography was considered the preferred confirmatory modality. Telephone interviews were used only to supplement clinical follow-up information, including symptoms, additional treatments, or survival status, and were not used to determine radiological obliteration. Patients without documented radiological obliteration were censored at the date of their last available imaging assessment. Secondary outcomes included intracranial bleeding, headaches, epilepsy, and neurological deficits at clinical presentation and after SRS. Sociodemographic, bAVM, and treatment features were analyzed using descriptive statistics, measures of central tendency and dispersion, and proportions.

Radiosurgery planning and treatment technique

**Figure 1:**
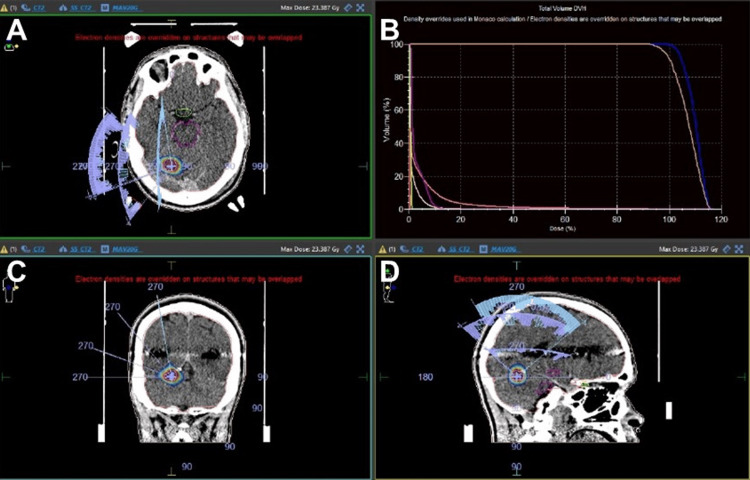
Planning of a LINAC-based SRS using triple image fusion for an occipital bAVM (A) Axial plane. (B) Dose-volume histogram. (C) Coronal plane. (D) Sagittal plane. SRS: stereotactic radiosurgery, bAVM: brain arteriovenous malformation, LINAC: linear accelerator

SRS was performed using an ELEKTA Axesse LINAC (Elekta AB, Stockholm, Sweden). After local or general anesthesia, a stereotactic frame was fixed to the patients’ heads. Subsequently, the patients underwent stereotactic angiography, gadolinium-enhanced brain MRI (using T1-weighted and FLAIR sequences to define the target), and contrast-enhanced head CT. Angiographic images were processed with ERGO++ (Elekta AB, Stockholm, Sweden), then integrated and fused with CT and MRI into the Monaco software for treatment planning (Figure 1).

Radiosurgery doses and prescribed isodoses were tailored to the maximum diameter of the bAVM nidus, its location, previous treatments, the eloquence of surrounding tissue, the proximity of surrounding organs at risk, and clinical experience. Doses ranged from 14 Gy (large/eloquent lesions) to 24 Gy (small, non-eloquent). Most patients received treatment in a single session; some underwent two-stage SRS sessions, with the second six months later. The highest marginal dose with the least risk of necrosis or injury ranged from 14-15 Gy in previously irradiated patients or eloquent territory locations to 23 Gy in previously non-irradiated patients with small lesions in non-eloquent territories. The dose was specified at the isocenter, and the entire nidus, as visualized by angiography and MRI, was defined as the target volume. The dose prescription was adjusted based on the proximity of the organs at risk to the target volume. All plans ensured 95% of the nidus received the prescribed dose.

Statistical analysis

Means were reported for variables with parametric distribution, while medians were used for variables with non-parametric distribution, including measures of dispersion. Percentages were used for categorical variables. Kaplan-Meier estimates were used to describe time to radiological obliteration, and survival curves were compared using the log-rank test. Two patients who died before completing follow-up were censored at the time of death. As a sensitivity analysis, the Kaplan-Meier estimator was compared with the cumulative incidence function derived from a Fine-Gray competing risks model that accounts for death as a competing event; both estimators were similar, with an absolute difference of approximately 2% (see supplementary material ). Because the median follow-up was 16.3 months and loss to follow-up was substantial, actuarial obliteration estimates at later time points were considered exploratory and interpreted with caution. The number of patients at risk was reported for all Kaplan-Meier curves.

Factors associated with obliteration were analyzed using univariable Cox proportional hazards models. The proportional hazards assumption was assessed using Schoenfeld residuals, with no violations detected. Results are presented as hazard ratios (HRs) with corresponding 95% confidence intervals (CIs) and p-values. Multivariable Cox regression was not performed because the number of observed obliteration events (n = 27) was insufficient to support stable multivariable estimation without overfitting. Univariable Cox proportional hazards models were therefore used only as exploratory analyses. Variables with small subgroup sizes or without a plausible biological relationship to obliteration, such as geographic region of residence, were not interpreted as independent predictors. Given the limited number of radiological obliteration events, multivariable modeling was restricted to avoid overfitting. Limited sensitivity analyses in multivariable Cox models were performed using clinically relevant covariates. A two-variable model included radiosurgery dose and SM grade. A second model, based on reviewer-suggested potential confounders, included bAVM size, SM grade, and prior embolization. The proportional hazards assumption was evaluated using Schoenfeld residuals. Complete-case analysis was used for multivariable models involving covariates with missing values. Because death before obliteration may act as a competing event, a competing-risk sensitivity analysis was performed by comparing Kaplan-Meier estimates with cumulative incidence estimates, treating death as a competing event.

For the dose-obliteration analysis, patients who underwent a second volume-staged SRS session were included in the main model, with the first-session prescribed dose used. A sensitivity analysis was performed in two ways: first, excluding all staged patients; and second, using the cumulative dose, defined as the sum of prescribed doses across sessions, for staged patients. All statistical analyses were conducted using R version 4.5.0 (R Foundation for Statistical Computing, Vienna, Austria, https://www.R-project.org/).

## Results

Patient and bAVM characteristics

A total of 82 patients who underwent SRS with triple-image fusion at our institution were included in the study. As shown in Table 1, the median age at the time of SRS was 43.5 years, and 51.2% of patients were female. Remarkably, a significant proportion (27, 33%) of patients in our sample resided outside Bogota, the city where our treatment center is located. Among these patients, only 10 (37%) completed the three-year follow-up. Regarding clinical presentation of the bAVMs, 59.8% of participants reported headaches, with a smaller fraction presenting with focal neurological deficits or seizures. Turning to the bAVM characteristics, almost half (44%) of patients presented with a ruptured bAVM at diagnosis. bAVMs were located primarily in the frontal lobe, accounting for 25.6% of the cohort. The temporal lobe was the most common anatomical location of the bAVM in those who completed follow-up (27.8%). Notably, the majority of bAVMs were situated in eloquent brain areas (74.4%) and were SM grade III (42.7%). No patients in our cohort had an SM grade V bAVM. The median size of bAVMs was 2.2 cm, with 46.3% having deep venous drainage and 19.5% having associated aneurysms.

**Table 1:** Patient demographics, bAVMs, and SRS characteristics * For AVMs with more than one location, the caudal location is expressed. ** The dose reported is the dose at the margin. Data are presented as the number of patients (%) unless indicated otherwise. Percentages correspond to column percentages. AVM: arteriovenous malformation, IQR: interquartile range, SRS: stereotactic radiosurgery, bAVMs: brain arteriovenous malformations, SM: Spetzler-Martin

Variable	Satisfactory 3-year follow-up (n = 36, 43.9%)	Unsatisfactory 3-year follow-up (n = 46, 56.1%)	Total (n = 82)
Median age in years (IQR)	36.0 (25.7-51.2)	44.5 (29.2-56.0)	43.5 (27.2-53.7)
Sex			
Female	19 (52.7)	23 (50)	42 (51.2)
Male	17 (47)	23 (50)	40 (48.8)
Region of residence			
Bogota	26 (72.2)	29 (63.1)	55 (67.1)
Atlántica	2 (5.6)	3 (6.5)	5 (6.1)
Andina	8 (22.2)	10 (21.7)	18 (21.9)
Orinoquía	0 (0)	3 (6.5)	3 (3.6)
Amazonía	0 (0)	1 (2.2)	1 (1.3)
Clinical presentation			
Seizures	10 (27.8)	10 (21.7)	20 (24.4)
Focal neurological deficit	12 (33.3)	17 (36.9)	29 (35.4)
Headache	21 (58.3)	28 (60.9)	49 (59.8)
Loss of coordination	4 (11.1)	3 (6.5)	7 (8.5)
AVM characteristics			
Ruptured (hemorrhage)	13 (36.1)	23 (50)	36 (44.0)
Median size in cm (IQR)	2.5 (1.4-3.1)	2.0 (1.2-3.5)	2.2 (1.2-3.5)
Anatomical location*			
Frontal lobe	8 (22.2)	13 (28.3)	21 (25.6)
Parietal lobe	9 (27.8)	10 (19.6)	19 (23.2)
Temporal lobe	10 (27.8)	5 (10.9)	15 (18.3)
Occipital lobe	6 (16.7)	9 (19.6)	15 (18.3)
Cerebellum	3 (8.3)	10 (21.7)	13 (15.9)
Basal ganglia	1 (2.8)	2 (4.3)	3 (3.6)
Brainstem	5 (13.9)	7 (15.2)	12 (14.6)
Eloquent area	28 (77.8)	33 (71.7)	61 (74.4)
SM score			
I	2 (5.5)	5 (10.9)	7 (8.5)
II	14 (38.9)	16 (34.8)	30 (36.6)
III	15 (41.7)	20 (43.4)	35 (42.7)
IV	5 (13.9)	5 (10.9)	10 (12.2)
Deep venous drainage	17 (47.2)	21 (45.6)	38 (46.3)
Associated aneurysm	9 (25.7)	7 (15.2)	16 (19.5)
Treatment variables			
Prior embolization	24 (66.7)	23 (50)	47 (57.3)
Median radiosurgery dose in Gy (IQR)**	20 (18-20)	20 (18-20)	20 (18-20)
Radiosurgery dose			
<19 Gy	10 (27.8)	11 (23.9)	21 (25.6)
19 Gy	7 (19.4)	14 (30.4)	21 (25.6)
20 Gy	9 (25)	11 (23.9)	20 (24.4)
>20 Gy	10 (27.8)	10 (21.7)	20 (24.4)
Second volume-staged radiosurgery	7 (19.4)	4 (8.7)	11 (13.4)

Treatment variables

Concerning treatment variables (Table 1), more than half of our patient cohort had undergone prior embolization (57.3%), and the median radiosurgery dose was 20 Gy (interquartile range (IQR) 18-20); 13.4% of patients underwent a second volume-staged radiosurgery session.

Follow-up

Adequate three-year institutional follow-up was available for 36 patients (43.9%). Among these, 27 achieved radiological obliteration, and nine remained without documented obliteration at three years. The remaining 46 patients (56.1%) were lost to institutional follow-up before three years and were censored on the date of the last available follow-up. Two patients died within three years due to causes associated with their bAVMs and were censored in the Kaplan-Meier analysis. Given the median follow-up of 16.3 months, actuarial estimates beyond 24 months should be interpreted cautiously.

Outcomes

**Figure 2:**
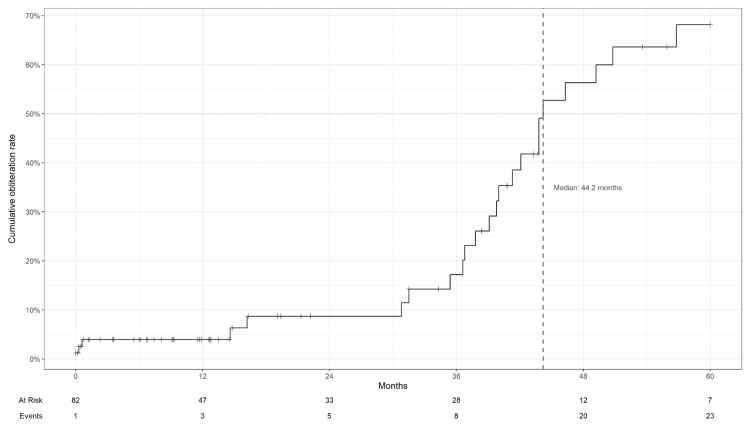
Five-year radiological obliteration outcomes after LINAC-based SRS for bAVMs Censored observations are indicated with + on the Kaplan-Meier curves. LINAC: linear accelerator, SRS: stereotactic radiosurgery, bAVMs: brain arteriovenous malformations

Complete radiological obliteration was documented in 27 patients (33.0%) during available follow-up. Obliteration was confirmed by angiography in 16 cases (59.3%) and by MRI in 11 cases (40.7%). The median follow-up was 16.3 months. Kaplan-Meier actuarial obliteration estimates were calculated at predefined time points; however, because of substantial censoring and the limited number of patients remaining under observation at later time points, estimates beyond 24 months were considered exploratory (Table 2, Figure 2).

**Table 2:** Radiological actuarial obliteration rate CI: confidence interval, SRS: stereotactic radiosurgery

Time after SRS (months)	Actuarial obliteration rate (%)	95% CI
12	4	0-8.3
24	8.7	0.7-16
36	17.2	4.9-27.9
48	56.3	34.9-70.7
60	68.2	45.3-81.5

**Figure 3:**
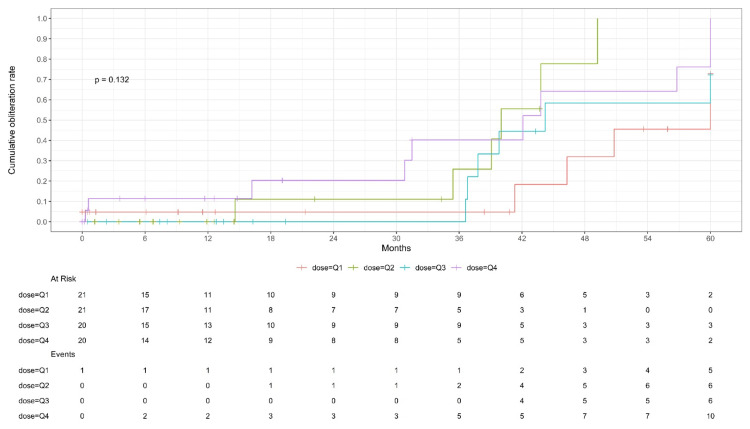
Exploratory actuarial radiological obliteration stratified by SRS dose categories. Q1 < 19 Gy, Q2 = 19 Gy, Q3 = 20 Gy, and Q4 > 20 Gy. Censored observations are indicated with + on the Kaplan-Meier curves. SRS: stereotactic radiosurgery

Kaplan-Meier analysis revealed no statistically significant differences (log-rank p = 0.132) across dose quartiles. The median time to obliteration was 40.0 months (95% CI: ≥35.4) for Q2, 42.1 months (95% CI: ≥30.8) for Q4, and 44.2 months (95% CI: ≥37.8) for Q3. Q1 showed the longest median time (60.0 months; 95% CI: ≥46.3) with the fewest obliteration events (5/21, 24%) (Figure 3).

Six post-SRS hemorrhages occurred, causing two deaths, as mentioned above. During available post-SRS follow-up, 20 patients had headaches, 14 experienced focal neurological deficits, and 11 reported seizures (Table 3).

**Table 3:** Symptomatic outcomes in obliteration of bAVM cases Data are presented as the number of patients (%) unless indicated otherwise. Percentages correspond to column percentages. bAVM: brain arteriovenous malformation

Variable	bAVM obliteration (n = 27)
Headache	
Resolution	9 (33.3)
Persistence	6 (22.2)
De novo	5 (18.5)
Asymptomatic	7 (25.9)
Seizures	
Resolution	3 (11.1)
Persistence	4 (14.8)
De novo	4 (14.8)
Asymptomatic	16 (59.3)
Focal neurological deficit	
Resolution	11 (40.7)
Persistence	1 (3.70)
De novo	2 (7.41)
Asymptomatic	13 (48.1)

Sensitivity analyses demonstrated that HR for obliteration was similar when excluding patients with second volume-staged radiosurgery (HR = 1.36, 95% CI 0.99-1.87, p = 0.058) or when considering only the first-session dose (HR = 1.35, 95% CI 0.98-1.86, p = 0.061), whereas using the cumulative dose across all sessions did not show a meaningful association (HR = 0.99, 95% CI 0.94-1.03, p = 0.616). Therefore, staged and non-staged patients were analyzed as a single cohort using the first-session dose.

Factors associated with bAVM obliteration

As depicted in Table 4, in univariate Cox analysis, the highest dose quartile (>20 Gy) (HR = 4.90; 95% CI 1.64-14.60; p = 0.004) and the presence of a focal deficit at diagnosis (HR = 2.29; 95% CI 1.04-5.01; p = 0.039) were significantly associated with an increased obliteration rate. Variables including numeric radiosurgery dose (HR = 1.35; 95% CI 0.99-1.87; p = 0.061) and loss of coordination at clinical presentation (HR = 2.74; 95% CI 0.94-8.02; p = 0.065) showed a trend towards association but did not reach statistical significance. In a limited multivariable Cox model including radiosurgery dose and SM grade, radiosurgery dose showed a trend toward higher obliteration rate (HR 1.38, 95% CI 0.96-1.97, p = 0.079), whereas SM grade was not significantly associated with obliteration (HR 1.25, 95% CI 0.68-2.30, p = 0.475). The model concordance was 0.57, and the proportional hazards assumption was not violated by Schoenfeld residual testing (global p = 0.59). A second multivariable Cox model including bAVM size, SM grade, and prior embolization showed no significant association for any of these variables. However, prior embolization violated the proportional hazards assumption, as indicated by Schoenfeld residual testing (p = 0.003; global p = 0.018), limiting the interpretability of the standard Cox model. In a competing-risk sensitivity analysis that treated death before obliteration as a competing event, cumulative incidence estimates were nearly identical to Kaplan-Meier estimates, with an absolute difference of approximately 2% at 60 months. Given the small number of competing events, Kaplan-Meier estimates were retained as the primary descriptive analysis and interpreted with caution (see supplementary material).

**Table 4:** Univariable Cox proportional hazards models: associated factors for complete obliteration of arteriovenous malformations Statistically significant values (p < 0.05) are highlighted. P-values >0.05 and <0.10 were considered indicative of a trend. Geographic region of residence was not included in the main predictor analysis because it lacks a direct biological rationale for obliteration and was considered more likely to reflect follow-up access, referral patterns, and unmeasured confounding. bAVM: brain arteriovenous malformation, CI: confidence interval, HR: hazard ratio, SM: Spetzler-Martin, NA: not applicable

Variable	HR	95% CI	p-value
Sociodemographic data			
Age (numeric)	1.01	0.99-1.03	0.34
Sex			
Male	Ref.	-	-
Female	1.37	0.64-2.93	0.42
Clinical presentation			
Seizures	0.78	0.33-1.84	0.57
Focal neurological deficit	2.29	1.04-5.01	0.039
Headache	0.82	0.39-1.76	0.61
Loss of coordination	2.74	0.94-8.02	0.06
bAVM characteristics			
Ruptured (hemorrhage)	1.32	0.61-2.85	0.48
Size in cm (numeric)	0.85	0.63-1.14	0.27
Anatomical location			
Frontal lobe	1.43	0.46-24.44	0.24
Parietal lobe	0.94	0.63-31.29	0.13
Temporal lobe	0.61	0.28-17.63	0.45
Occipital lobe	1.13	0.28-17.63	0.45
Cerebellum	0.93	0.15-18.87	0.68
Basal ganglia	>10	NA	0.99
Brainstem	0.91	0.31-2.63	0.86
SM score			
I	Ref.	-	-
II	0.78	0.17-3.53	0.74
III	0.75	0.17-3.41	0.71
IV	0.63	0.10-3.81	0.61
Deep venous drainage	0.93	0.43-1.99	0.85
Associated aneurysm	1.20	0.50-2.85	0.68
Treatment variables			
Prior embolization	1.78	0.78-4.08	0.16
Radiosurgery dose (numeric)	1.36	0.99-1.86	0.06
Radiosurgery dose (categories)			
<19 Gy	Ref.	-	-
19 Gy	2.48	0.75-8.24	0.13
20 Gy	1.48	0.42-4.62	0.57
>20 Gy	4.90	1.62-14.8	0.004
Second volume-staged radiosurgery	0.56	0.19-1.64	0.29

## Discussion

Main findings

In this retrospective single-institution cohort, complete radiological obliteration after LINAC-based SRS was documented in 27 of 82 patients (33.0%) during available follow-up. Although actuarial obliteration estimates increased over time, these estimates should be interpreted with caution because the median follow-up was 16.3 months, and more than half of the cohort discontinued institutional follow-up before three years. Therefore, our findings should be viewed as preliminary observational data describing outcomes and follow-up challenges in a Latin American quaternary care setting, rather than as definitive evidence of long-term effectiveness. Prior reports on LINAC-based SRS have reported three-year obliteration rates of 60% [33], 72% [34], and 74.5% [27]. Matsuo et al. [35] reported that rates during the first three years initially appeared lower, yet after a 15-year follow-up, comparable values were eventually attained. Thenier-Villa et al. [29], which included patients with similar baseline characteristics (bAVM rupture status, deep venous drainage, size, and location) and a fifteen-year follow-up, also showed a similar obliteration rate. Although prior LINAC-based SRS series have reported three-year obliteration rates ranging from approximately 60% to 75%, direct comparison with our cohort is limited by differences in follow-up duration, censoring, imaging confirmation, patient selection, and study design. Previous studies from high-income countries emphasize access to the full spectrum of SRS technologies, which is not a reality in most Latin American countries and healthcare settings.

The high rates of eloquent area involvement and ruptured bAVMs at diagnosis align with global reports. Our cohort received slightly higher median SRS doses than those reported at other centers [33], and post-treatment hemorrhage rates were comparable [29,33,35]. Our exploratory univariable Cox analysis was consistent with a possible dose-response relationship reported in the literature. However, this finding should be interpreted with caution, given the limited number of events and the absence of definitive multivariable adjustment [3,4,33], underscoring the importance of precise dose planning.

Disruption in continuity of healthcare: a major challenge in Latin America

One of the major challenges identified in our study was the high rate of loss to follow-up, particularly among patients living outside the city where our center is located. This problem, common throughout Colombia, likely stems from the fragmented nature of the national healthcare system. As described by Caicedo et al. and Guerrero et al., government-funded health insurance agencies (EPS, for its Spanish acronym) contract with both private and public healthcare institutions, aiming to cover the largest possible population at the lowest cost [36,37]. Consequently, many radiotherapy centers are forced to prioritize patient volume over quality and become heavily dependent on EPS contracts. This fragmented system leads to multiple administrative barriers, such as abrupt contract terminations, which often compel patients to change providers and undergo prolonged insurance authorization processes, particularly within the subsidized insurance system, thereby complicating multidisciplinary cancer care and follow-up [36].

Furthermore, the fragmentation of oncologic services in Colombia is exacerbated by the nationwide shortage of radiation oncology professionals and of other medical specialists [36,38]. Radiotherapy centers are also relatively scarce and are mainly concentrated in major urban areas, requiring patients from marginal regions to undertake long and expensive journeys [36]. These burdens may decrease patient satisfaction with the care they receive [37] and ultimately lead patients to discontinue their cancer treatment [36]. In this context, the geographic region of residence should be interpreted as a marker of potential barriers to longitudinal follow-up rather than as a determinant of biological response to SRS.

Despite loss to follow-up, our 36-patient sample compares favorably with other Colombian and regional studies [16,39]. A significant proportion of Latin American countries, including Colombia, operate under resource-constrained healthcare systems that limit access to high-cost technologies such as Gamma Knife: in 2016, whereas Japan, the United States of America, Australia, and the United Kingdom had ratios ranging from approximately 2.4 to 8.2 million people per Gamma Knife device, South American countries like Brazil and Colombia had 103.8 and 24.3 million people per device, respectively [38]. As a result, LINAC-based SRS becomes a more practical alternative, offering similar obliteration rates safely and cost-effectively [38]. In regions where repeated or advanced treatments are not readily available, optimizing dose delivery is critical to maximizing the efficacy of bAVM management [3,4,33]. This situation highlights the urgent need for more robust healthcare infrastructure and consistent patient follow-up protocols, which are critical to improving long-term outcomes.

Strengths

Our study has several strengths. First, during SRS planning, we used triple-fusion imaging (angiography, MRI, and CT), which enhances the precision of bAVM targeting. Second, our study provides new insights into the treatment of bAVMs with LINAC-based SRS in a limited-resource scenario with lower access to advanced technologies such as Gamma Knife or CyberKnife [38]. This aspect differentiates our findings from the studies conducted in higher-income regions with widely available cutting-edge technologies [4,5,7,11,14,22,28]. Finally, our study addresses a critical gap in the literature by presenting institutional outcome data from a Latin American context, where published experience with LINAC-based SRS for bAVMs remains limited and underrepresented in global bAVM research. The use of time-to-event analysis allowed exploratory description of obliteration over time, although the estimates are limited by censoring and incomplete follow-up. The collaboration between multiple disciplines (radiation oncologists, neurosurgeons, and medical physicists) adds robustness and clinical relevance to our results.

Limitations

Some limitations must be acknowledged. First, this was a retrospective single-institution study, which introduces potential selection bias and limits the generalizability of our findings. The sample size was relatively small, with only 27 obliteration events, which limited statistical power and resulted in wide CIs for HR estimates. Additionally, several categorical variables had few observations in certain subgroups, reducing precision and increasing the risk of unstable estimates.

A major limitation is the relatively short median follow-up of 16.3 months in the context of bAVM radiosurgery, where radiological obliteration may occur several years after treatment. Although Kaplan-Meier methods were used to estimate actuarial obliteration over time, the substantial loss to follow-up before three years limits the robustness of later estimates. Therefore, actuarial obliteration rates at later time points should be considered exploratory and interpreted with caution, as they may be affected by censoring and attrition bias. Although deaths were treated as censored events in the primary analysis, a competing-risks sensitivity analysis showed negligible differences between the two estimators, given the low number of deaths (n = 2).

The main analyses were univariable and did not adjust for potential confounders. Consequently, observed associations should be interpreted as exploratory rather than causal. In particular, the geographic region of residence should not be interpreted as a biologically meaningful predictor of obliteration. Small subgroup sizes, referral patterns, access to follow-up imaging, healthcare system fragmentation, or other unmeasured confounding factors likely influence any apparent association between geographic origin and obliteration.

For patients treated with staged radiosurgery, the cumulative dose was calculated as a simple sum of prescribed doses, which may not fully reflect true biological exposure. Additionally, the evaluation of symptomatic outcomes directly related to radiation toxicity was limited by the absence of systematic data on radiographic radiation injury and treatments for such toxicity. Finally, we did not collect complete data on salvage treatments, such as surgery or embolization, after initial SRS, which limits our ability to fully assess post-treatment management strategies and long-term outcomes.

Future insights

Future efforts should focus on addressing the challenges of patient follow-up through telemedicine initiatives and regional hospital networks. Such approaches could help bridge the gap between central treatment centers and rural areas. Standardized follow-up protocols could help ensure that bAVM patients in resource-limited regions receive the continuous care required for accurate long-term outcome evaluation. Future prospective studies with larger patient populations and rigorous follow-up protocols are needed to better evaluate the outcomes of SRS for bAVMs.

## Conclusions

This retrospective, single-institution study describes preliminary radiological and clinical outcomes following LINAC-based SRS for bAVMs at a Latin American quaternary care center. Complete obliteration was documented in a subset of patients during available follow-up, and a higher prescription dose showed an exploratory association with a shorter time to obliteration. However, the short median follow-up, substantial loss to follow-up, absence of a comparator group, and lack of multivariable adjustment limit the ability to draw definitive conclusions regarding effectiveness. Geographic origin should be interpreted as a potential marker of access-to-care and follow-up barriers rather than as a biological predictor of obliteration. Prospective multicenter studies with standardized long-term imaging follow-up are needed.

## Figures and Tables

**Figure 4:**
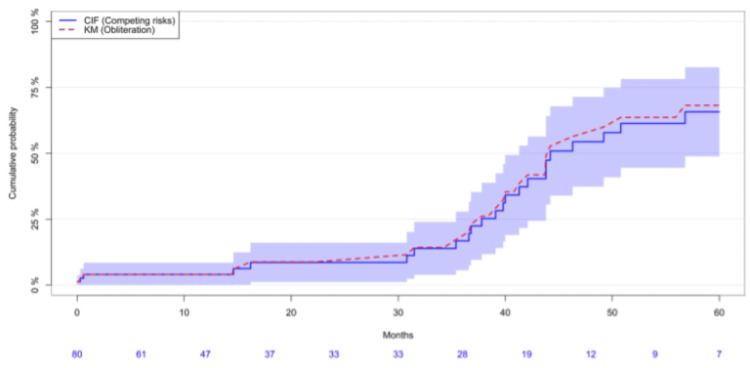
Sensitivity analysis comparing Kaplan-Meier and cumulative incidence function estimates

## Appendices

Sensitivity analysis

As a sensitivity analysis, the Kaplan-Meier estimator was compared with the cumulative incidence function derived from a Fine-Gray competing risks model; both estimators were virtually superimposable, with an absolute difference of approximately 2% at 60 months (Figure 4).
